# Development, cross-cultural adaptation, and validation of the Persian Mississippi Aphasia Screening Test in patients with post-stroke aphasia

**Published:** 2015-04-04

**Authors:** Ahmad Reza Khatoonabadi, Noureddin Nakhostin-Ansari, Amin Piran, Hamid Tahmasian

**Affiliations:** 1 Department of Speech Therapy, School of Rehabilitation, Tehran University of Medical Sciences, Tehran, Iran; 2 Department of Physiotherapy, School of Rehabilitation, Tehran University of Medical Sciences, Tehran, Iran

**Keywords:** Stroke, Aphasia, Mississippi Aphasia Screening Test

## Abstract

**Background: **The Mississippi Aphasia Screening Test (MAST) is a brief screening test for assessing the expressive and receptive language abilities in patients with aphasia. The objective of the study was to develop and validate the Persian version of the MAST (MASTp) as a screening test for language disorders in patients with post-stroke aphasia.

**Methods:** This study used a cross-sectional design to cross-culturally adapt the MASTp following the guidelines for the process of cross-cultural adaptation of measures. A total of 40 subjects (20 patients with post-stroke aphasia and 20 healthy subjects) were included. The MASTp was tested for floor or ceiling effects, internal consistency reliability, intra-rater reliability, discriminative validity, and factor structure.

**Results: **There were no floor or ceiling effects for MASTp total score. The MASTp yielded values for internal consistency reliability that were not adequate (Cronbach’s alpha 0.64 and 0.66 for test and retest, respectively. The intra-rater reliability of the MASTp within a 7 day-interval was excellent for total score (ICC _agreement_ = 0.96) and both expressive index (ICC = 0.95) and receptive index (ICC _agreement_ = 0.98). here were statistically significant differences in MASTp total scores and both indexes between patients and healthy subjects suggesting the discriminative validity of the MASTp (P < 0.001). Factor analysis revealed a 3-factor solution, which jointly accounted for 72.06% of the total variance. Additional factor analysis suggested 6-item MASTp as a unidimensional measure.

**Conclusion: **The MASTp is useful as a valid and reliable screening tool for evaluation of language abilities in Persian speaking patients with aphasia after stroke.

## Introduction

Aphasia is one of the most common and devastating consequences of stroke. It is reported that the aphasia is present in 21-38% of patients with acute stroke.^1^ A prospective, population-based study of the epidemiology of aphasia found that 43 of 100,000 inhabitants are affected per year from first ischemic stroke.^2^ The burden of aphasia is high. Aphasia is associated with higher mortality, morbidity, and worse functional outcomes.^1^^,^^3^ Communication problems in patients with post-stroke aphasia can impair their quality of life.

It is important to identify the aphasia early after stroke to maximize the therapy gain and to improve language outcomes. Screening assessment using tools with sound psychometric and administrative properties can provide a quick and efficient means to diagnose the presence of aphasia post-stroke. There are several screening instruments reported in the published stroke literature. The Mississippi Aphasia Screening Test (MAST) is one of the most valuable screening devices to identify the patients withaphasia.^4^

The MAST is a valid and repeatable screening tool for quickly measuring the expressive and receptive language abilities in patients with aphasia. The MAST has 9 subtests ranging from 1 to 10 items per sub-scale (naming, automatic speech, repetition, yes/no accuracy, object recognition from a field of five, following verbal instructions, reading instructions, verbal fluency, and writing/spelling to dictation).^5^ The scores from each item of MAST are summed to produce sub-scale scores (receptive and expressive, each range 0-50). The scores from both sub-scales are summed to provide total score (range 0-100). The MAST is a simple and brief measure, and time required to administer the MAST is ~5-15 min.^5^

The MAST is developed and validated in English language. To be used in different languages other than English, the MAST is required to be translated and cross-culturally adapted to ensure that the translated version is appropriate and relevant in the target language. The translation and validation process following standard guidelines tries to produce the equivalency of the source MAST conceptually and semantically and the target language.^6^ Although the MAST is translated into Czech,^7^ Spanish,^8^ and Telugu language,^9^ no Persian version exists.

Therefore, the aim of the present study was to translate and cross-culturally adapt the MAST into Persian language (MASTp). The floor or ceiling effects, internal consistency reliability, intra-rater reliability, discriminative validity, and factor structure were examined.

## Materials and Methods

A cross-sectional study was used to develop and cross-culturally adapt the MASTp, and to assess the reliability and validity of the MASTp. The study design was approved by the Review Board, School of Rehabilitation, and the Ethical Committee of Tehran University of Medical Sciences, Iran.

The translation of the MASTp was performed following proposed guideline by Beaton et al.^6^ Two bilingual translators whose native language was Persian independently forward-translated the MAST into Persian, and another two bilingual translators whose native language was English independently back-translated the synthesized Persian version into the English. An expert committee reviewed the all documents and produced the pre-final version. Ten speech-language pathologists (SLP) expert in aphasia therapy were invited to evaluate the pre-final version of the MASTp to give their comments on the clarity and meaningful of the translation. The feedbacks from the experts were reviewed by the committee, and some proposed changes were applied to produce the final MASTp (sub-scale of Verbal fluency: “knowledge is power” was substituted for “three strikes”; sub-scale of Repetition: “table” was substituted for “pot”; sub-scale of writing to dictation: “go” and “machine” was substituted for “sit” and “airplane”, respectively. The final MASTp is shown in Appendix.

Patients were included with the following inclusion criteria: (1) age 18-65 years; (2) first-ever stroke resulted in aphasia; (3) stroke duration of at least 1 month; (4) able to read and write Persian language. Patients with severe visual/auditory and cognitive deficits were excluded. Healthy and neurologically intact subjects were also included. All participants agreed and signed written informed consent prior to participate in the study.

Patients were tested by an experienced SLP familiar with the MASTp. The SPL administered the MASTp in all patients and healthy subjects. Patients were tested again with 1-week interval for intra-rater reliability.^10^ The Edinburgh inventory Laterality was used to assess the handedness in all subjects.^11^

Kolmogorov-Smirnov test was performed to assess whether continuous data have a normal distribution. Demographic characteristics were compared between groups using the independent *t *test (continuous data) or Mann–Whitney U-Test (categorical data). The independent *t *test was applied to estimate the discriminative validity by comparing MASTp scores between patients and healthy subjects. The Cronbach’s alpha statistic was used to calculate internal consistency reliability. The Cronbach’s alpha between 0.7 and 0.95 was considered high.^10^ To measure intra-rater reliability, the intraclass correlation coefficient (agreement) (ICC_agreement_) (two-way random effects model, single measure) was calculated. A minimum of 0.7 was regarded for reliability. An ICC coefficient of more than 0.75 was interpreted excellent reliability; 0.60-0.75, good reliability; and 0.40-0.59, fair reliability.

The percentage frequency of lowest or highest possible score achieved by subjects were calculated as floor or ceiling effect. The floor or ceiling effects > 15% were considered to be significant. The data were analyzed using the SPSS software (version 18.0, SPSS, Inc., Chicago, IL, USA). An alpha of < 0.05 was considered as statistically significant.

## Results

All the continuous variables were normally distributed. In this study, 20 patients [13 male and 7 female; mean age ± standard deviation (SD) = 52.3 ± 8.2 years, range = 36-65] and 20 healthy subjects (10 male and 10 female; mean age ± SD = 49.6 ± 8.8 years, range = 27-65) were participated. The mean education ± SD in patients and healthy subjects was 11.2 ± 5.5 years (range = 1-18) and 10.3 ± 4.0 years (range = 5-18), respectively. Eighteen patients (90%) and 19 healthy subjects (95%) were right handed. There were no significant differences between 2 groups for age (P = 0.210), education (P = 0.560), gender (P = 0.340), and laterality (P = 0.550). Duration since stroke in patients group was 27.2 ± 50.75 months (range 1-224).

***Floor or ceiling effects***

Floor or ceiling effects were not seen for MASTp total score in test (44.60 ± 16.11, range = 6-70) and retest (46.0 ± 16.14, range = 6-67).No patients were scored the lowest or highest possible score on MASTp.

***Discriminative validity***

There was a statistically significant difference in MASTp total scores (Levenes’ test for equality of variances: F = 25.32, P < 0.001; t = −14.80, df = 19.49, P < 0.001), expressive index scores (Levenes’ test for equality of variances: F = 44.57, P < 0.001; t = −14.41, df = 19.94, P < 0.001, and receptive index scores between the 2 groups (Levenes’ test for equality of variances: F = 23.84, P < 0.001; t = −9.49, degree of freedom (df) = 19.46, P < 0.001) (Table 1).

***Internal consistency***

The Cronbach’s alpha was 0.64 for test, and the Cronbach’s alpha if item deleted ranged between 0.52 and 0.72 (Table 2). For retest, the Cronbach’s alpha was 0.66, and the Cronbach’s alpha if item deleted ranged between 0.56 and 0.73 (Table 2).

***Intra-rater reliability***

The ICC _agreement_ for the intra-rater reliability of the MASTp total score was excellent (0.96, 95% CI = 0.90-0.98, P < 0.001). The intra-rater reliability for the expressive MASTp (0.95, 95% CI = 0.88-0.98, P < 0.001) and receptive MASTp (0.98, 95% CI = 0.94-0.99) delivered excellent results. 

**Table 1:** Mean ± standard deviation of Mississippi Aphasia Screening Test (MAST) scores by group for test (n = 20)

**MAST scale**	**Healthy subjects (Mean ± SD)**	**Patients (Mean ± SD)**	**P**
Naming	9.09 **± **0.44	3.90 ± 4.27	< 0.001
Automatic speech	9.07 **±** 0.73	4.45 ± 3.64	< 0.001
Repetition	9.09 **± **0.47	4.20 ± 2.96	< 0.001
Yes/No responses	19.08 ± 0.61	13.03 ± 6.16	< 0.001
Object recognition	10.00 ± 0.00	9.00 ± 2.55	0.080^٭^
Following instructions	10.00 ± 0.00	1.01 ± 2.63	<0.001
Reading instructions	9.04 ± 0.94	5.08 ± 2.50	< 0.001
Verbal fluency dictation	9.75 ± 1.11	0.75 ± 1.83	< 0.001
Writing/spelling	9.08 ± 0.89	2.10 ± 3.21	< 0.001
Expressive index	49.15 ± 1.63	15.40 ± 10.34	< 0.001
Receptive index	49.01 ± 1.02	29.20 ± 9.32	< 0.001
Total score	98.25 ± 1.83	44.60 ± 16.11	< 0.001

٭ Not significant; MAST: Mississippi Aphasia Screening Test; SD: Standard deviation

**Table 2:** Cronbach’s alpha if item deleted for Mississippi Aphasia Screening Test (MAST)

**Subtests**	**Scale mean if item deleted**		**Scale variance if item deleted**		**Corrected item-total correlation**		**Squared multiple correlation**		**Cronbach’s Alpha if item deleted**	
	**Test**	**Retest**	**Test**	**Retest**	**Test**	**Retest**	**Test**	**Retest**	**Test**	**Retest**
Naming	40.70	42.00	171.91	188.00	0.62	0.56	0.87	0.87	0.52	0.57
Automatic speech	40.15	41.25	191.71	187.15	0.54	0.59	0.73	0.70	0.56	0.56
Repetition	40.40	41.90	201.10	196.62	0.59	0.63	0.76	0.76	0.56	0.57
Yes/No Responses	31.30	32.00	178.22	177.68	0.26	0.29	0.56	0.66	0.67	0.68
Object recognition	35.60	37.10	202.57	197.46	0.70	0.73	0.65	0.65	0.55	0.56
Following verbal instructions	38.80	40.00	241.85	237.47	0.15	0.24	0.55	0.57	0.64	0.65
Reading instructions	43.50	44.90	234.05	241.04	0.23	0.15	0.64	0.68	0.63	0.66
Verbal fluency dictation	43.85	45.25	236.03	239.04	0.36	0.32	0.57	0.57	0.62	0.64
Writing/spelling	42.50	43.60	271.74	266.78	-0.21	-0.16	0.53	0.57	0.72	0.73

***Factor structure***

**Figure 1:**
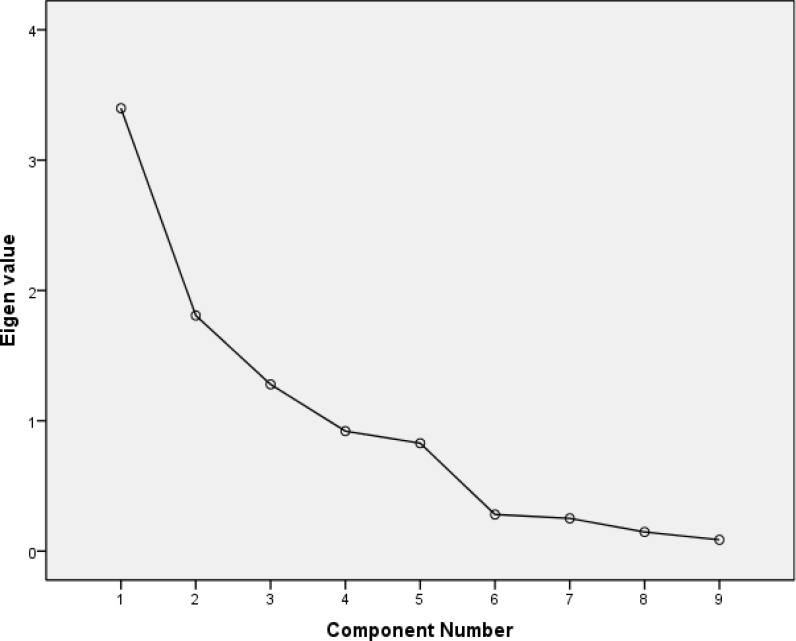
Scree plot for Persian Mississippi Aphasia Screening Test shows three latent factors

The Kaiser-Meyer-Olkin (KMO) measure of sampling adequacy was 0.56. Bartlett’s test of sphericity was 79.15 (P < 0.001). A principal component analysis with varimax rotation loaded 3 latent factors with Eigen values greater than 1, which jointly accounted for 72.06% of the total variance. The first factor (expressive) included 6 items, which explained 37.76% of the total variance (Eigen value = 3.40). The second factor (receptive) included 3 items, which explained 20.09% of the total variance (Eigen value = 1.81). The third factor (writing) included 2 items, which explained 14.22% of the total variance (Eigen value = 1.28). The item of “Object recognition” was loaded with all factors but slightly more on the first factor. The results are illustrated in table 3. The scree plot for MASTp is shown in figure 1.

**Figure 2:**
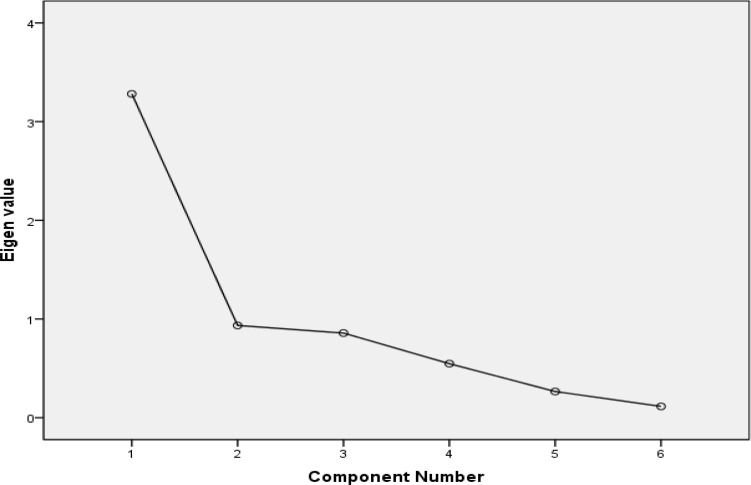
Scree plot for 6-item Persian Mississippi Aphasia Screening Test showing 1 homogenous factor

The Cronbach’s alpha for the extracted factors were 0.83, 0.60, and 0.42, respectively. Since the alpha value was acceptable only for the first factor, we thus further proceeded to test it for the factor structure. A principal component analysis with varimax rotation produced 1 homogenous measure for 6-item scale, which explained 54.68% of the total variance (KMO = 0.66, Bartlett’s test = 50.60, P < 0.001, Eigen value = 3.28). Figure 2 shows the scree plot for 6-item MASTp.

**Table 3:** The factor structure of the Mississippi Aphasia Screening Test (MAST)

**MASTp items**	**Factors**		
	**Expressive**	**Receptive**	**Writing**
Naming	0.884		
Automatic speech	0.878		
Repetition	0.848		
Yes/no responses		0.871	
Object recognition	0.549	0.509	0.516
Following verbal instructions		0.842	
Reading instructions	0.556		
Verbal fluency dictation	0.584		
Writing/spelling			0.865

MASTp: Persian Mississippi Aphasia Screening Test

## Discussion

This study presented the process of cross-cultural adaptation and validation of MAST for Persian speaking health professionals in particular SLPs to evaluate patients with post-stroke aphasia. The process of translation and cross-cultural adaptation in line with the translation versions of the MAST^7^^-^^9^ was performed without any difficulty and resulted in a measure in Persian language, the MASTp. The equivalency of MASTp with the original English version ensures that a study that uses the MASTp can compare the results with those that used the MAST in English as well as other languages. The standard methodology used in developing the MASTp ensured the face and content validity of this screening instrument. This study further demonstrated that the MASTp has discriminative validity, internal consistency reliability, and intra-rater reliability. As far as we know, this is the first validation study of the MASTp.

***Ceiling or floor effects***

In this study, no patients scored 0 or 100 on the MASTp total score, and the MASTp total scores were well distributed. The ceiling or floor effects were not reported for the English^5^ and the translated versions of the MAST.^7^^-^^9^ The lack of ceiling or floor effects further verifies the content validity of the MASTp.

***Discriminative validity***

As expected, patients scored poorly than the healthy subjects on all MASTp subtests as well as the total score, expressive index, and receptive index. Current findings are consistent with those of the original and translated versions of the MAST^5^^,^^7^^-^^9^ indicating the discriminative validity of the MASTp. This finding indicates that the MASTp was capable of discriminate between stroke patients with aphasia and healthy subjects.

The “Object recognition” subtest showed similar performance among patients with post-stroke aphasia and healthy subjects. This finding is in line with those of the Nagendar and Ravindra in the validation study of the Telugu language version of the MAST.^9^ The similar performance on the “Object recognition” among patients and healthy subjects may be explained by the fact that the “Object recognition” subtest depends primarily on visual-perceptual abilities.^5^

***Internal consistency reliability***

The internal consistency reliability indicates the interrelatedness among the items assessing how each item relates to the other items.^12^ Cronbach’s alpha in this study did not quite reach the cut-off score of 0.7 for acceptable internal consistency reliability. Cronbach’s alpha is not reported for the original English and translated versions of the MAST.^5^^,^^7^^-^^9^ One reason could be that the internal consistency for culturally adapted measures might be typical to be lower compared to the original tool. Another reason for the lower value found in this study could be the small number of patients. We noticed that when the item of “Writing” omitted the Cronbach’s alpha value improved, and the internal consistency reliability reached the acceptable level both for test (0.72) and retest (0.73). This suggests that the “Writing” might be redundant to the MASTp. The improvement of the internal consistency reliability with removing “Writing” indicates that the items of MASTp are not homogeneous and thus not measuring the same concept.^10^ A further study with larger sample size is needed to confirm the results.

***Intra-rater reliability***

The intra-rater reliability of the MASTp was excellent. A study to adapt the MAST to the Telugu language observed good inter-rater reliability and high test-retest reliability (r = 0.993).^9^ Kostalova et al. evaluated the inter-rater reliability of the Czech language version of the MAST and found acceptable inter-rater reliability of MAST total score.^7^ In another study to validate the MAST into Spanish language in patients with stroke, Authors reported excellent inter-observer reliability and test-retest reliability (ICC = 0.99).^8^ In our study, the period between the two administrations was 1 week to prevent recall and to ensure that clinical changes have not occurred.^10^ Excellent intra-rater reliability observed for the total MASTp as well as both the expressive and receptive indexes indicates that when the MASTp administered repeatedly by an examiner in stable stroke patients with aphasia can provide similar scores over time. Intra-rater reliability was not assessed for the original English and culturally adapted versions of the MAST.^5^^,^^7^^-^^9^

***Factor analysis***

Unidimensionality of a scale must be investigated with factor analysis to get an interpretable meaning for an internal consistency reliability statistic.^12^ In the current study, it was found that the MASTp was not unidimentional, and the factor analysis yielded 3-factor solution (Expressive, Receptive, and Writing). It was noted that the “Writing” item was appeared as an independent factor. The performance on the “Writing” item was poor in this sample of patients with post-stroke aphasia compared to the healthy subjects. The reason could be that the “Writing to dictation” requires intact left hemisphere that is dominant for this task for right handed people. In the current study, 90% of patients were right handed, and “Writing to Dictation” requires optimized motor performance of the hand. Hemiplegia or muscle paralysis on one side of the body is a common outcome after stroke, and voluntary movements need commands from the cortex to be transmitted to the peripheral neuromuscular system via the descending tracts. It has been documented that the voluntary activation is impaired bilaterally in the upper limb after stroke, and cortical connectivity on the more affected side is reduced.^13^ The Cronbach’s alpha for MASTp improved when the “Writing” item was removed; this finding together with the results of factor structure analysis suggests that the “Writing” item is redundant for the MASTp. Further study is suggested to clarify the current results. The “Object recognition” was loaded on all the 3 extracted factors. To clarify to which factor that the “Object recognition” is related, we conducted internal consistency reliability analysis for all the extracted factors. Cronbach’s alpha reached the acceptable level only for the first factor (Expressive subscale). Factor analysis for the first factor reduced the original 9-item scale to an 6-item scale and extracted 1 factor demonstrating the 6-item MASTp as a unidimensional screening instrument for patients with post-stroke aphasia. This finding indicates that when using MASTp for screening of patients with post-stroke aphasia, the 3 items of original MAST can be redundant (Writing, Yes/No responses, following instructions). The unidimensionality of the 6-item MASTp is an indication of construct validity. Factor analysis has not been performed to identify the possible latent subscales in previous studies with English and adapted versions of the MAST.^5^^,^^7^^-^^9^

***Limitations***

There are some limitations of the study, which have to be addressed. First, sample size of patients was small. At least 50 patients must be included in validation studies. Other psychometric characteristics such as inter-rater reliability, diagnostic accuracy of sensitivity and specificity, construct validity, responsiveness and changes over time with the MASTp will be necessary to be determined in future investigations.

## Conclusion

The Persian version of the MAST is a valid and reliable instrument to assess patients with post-stroke aphasia. The MASTp demonstrated face validity, content validity, discriminative validity, and intra-rater reliability. The psychometric properties of the MASTp suggest that this brief screening measure is appropriate for clinical and research studies in Persian speaking countries.

## Notes:

**How to cite this article: **Khatoonabadi AR, Nakhostin-Ansari N, Piran A, Tahmasian H. Development, cross-cultural adaptation, and validation of the Persian Mississippi Aphasia Screening Test in patients with post-stroke aphasia. Iran J Neurol 2015; 14(2): 101-7.
